# Electronic structure of MAPbI_3_ and MAPbCl_3_: importance of band alignment

**DOI:** 10.1038/s41598-019-50108-0

**Published:** 2019-10-22

**Authors:** Marco Caputo, Nicola Cefarin, Andrea Radivo, Nicola Demitri, Lara Gigli, Jasper R. Plaisier, Mirco Panighel, Giovanni Di Santo, Sacha Moretti, Angelo Giglia, Maurizio Polentarutti, Filippo De Angelis, Edoardo Mosconi, Paolo Umari, Massimo Tormen, Andrea Goldoni

**Affiliations:** 1 Elettra - Sincrotrone Trieste, s.s. 14 Km 163.5 in Area Science Park, Basovizza (Trieste) 34149, Trieste, Italy; 2 IOM-CNR Lab. TASC, s.s. 14 Km 163.5 in Area Science Park, Basovizza (Trieste) 34149, Trieste, Italy; 3 Dipartimento di Fisica - Università di Trieste, via Valerio Trieste, Trieste, Italy; 4 CNR - Institute of Atmospheric pollution Research - Sezione di Rende - c/o Polifunzionale - UNICAL 87036 Rende (CS), Rende, Italy; 5 Computational Laboratory for Hybrid/Organic Photovoltaics (CLHYO), CNR-ISTM, Via Elce di Sotto 8, I-06123 Perugia, Italy; 6 CompuNet, Istituto Italiano di Tecnologia, Via Morego 30, 16163 Genova, Italy; 7 Dipartimento di Fisica e Astronomia - Università di Padova, via Marzolo, 35131 Padova Italy

**Keywords:** Condensed-matter physics, Materials for devices, Materials for energy and catalysis, Electronic properties and materials

## Abstract

Since their first appearance, organic-inorganic perovskite absorbers have been capturing the attention of the scientific community. While high efficiency devices highlight the importance of band level alignment, very little is known on the origin of the strong n-doping character observed in the perovskite. Here, by means of a highly accurate photoemission study, we shed light on the energy alignment in perovskite-based devices. Our results suggest that the interaction with the substrate may be the driver for the observed doping in the perovskite samples.

## Introduction

Since the discovery of Methylammonium Lead Iodide (MAPbI_3_) perovskite^1^, a tremendous effort of the scientific community made was focused on exploiting this material as a very efficient light absorber. Initially, perovskite has been used as sensitizer for all-solid-state dye-sensitized solar cells (DSSCs)^2–5^, but soon it has been clear that perovskite could act also as charge transport medium exploiting its charge transport properties^2,4,6–9^. This approach opened the route to planar architecture devices that revolutionized the photovoltaic research scenario of the recent years^10–16^.

Beside the benchmark molecular system MAPbI_3_, several alternative organic/inorganic perovskites have been developed. First attempts to replace the environmental problematic Lead with Tin have been performed^17^, along with the substitution of metylammonium with formamidinium cationic molecule^18^. Also the counterion of the perovskite has been subjected to substitutions, leading to a fine tuning of the band gap^5,19^, and stability improvement^20^.

The most intriguing deviation from the initial MAPbI_3_ is represented by the mixed phase MAPbI_3−x_Cl_x_: here, a small part of the chlorine used in the PbCl_2_ precursor is supposed to become part of the final perovskite, thought it proves to be very difficult to observe. The difference in the two phases is evident in terms of charge recombination rate, sensibly lower in the mixed phase^4,7,21^. Initial studies indicated a co-existence of MAPbI_3_ and MAPbCl_3_ in a mixed phase, with a “chlorine rich” interface layer that facilitate the charge transfer between perovskite and TiO_2_^22,23^. Subsequent studies, however, demonstrated that only extremely small amount of chlorine, or none at all, remains in the final perovskite^24–28^, suggesting that the different device performance between the pure MAPbI_3_ and MAPbI_3−x_Cl_x_ is due to their film morphology, much more continuous in the latter case^29,30^.

Doping level, and hence band alignment, is an extremely important issue in organic-inorganic perovskite solar cells. Efficient devices need holes and electrons extracting layers with the correct energy level alignment^31,32^, therefore, understanding the details of the electronic structure of the perovskite absorber and the band alignment at the interfaces is of paramount importance. Initial experiments showed that both MAPbI_3_ and MAPbI_3−x_Cl_x_ are extremely n-doped semiconductor, with the Fermi level very close to the conduction band minimum. However, depositing the perovskite material on p-doped semiconductor, like PEDOT:PSS or NiO, results in a reduced n-doping for perovskite^33^ and, more in general, it has been shown that the perovskite electronic structure may change depending on the substrate used^34^. Wang *et al*. showed that even a self-doping effect is possible by changing the relative ratio PbI_2_/MAI in the precursor solution^35^. Thus, understanding the origin of this doping, and the way to control it, is fundamental in the smart design of perovskite-based heterojunction devices, representing the key to improve the efficiency of future devices.

To address all these open issues, we report an accurate electronic property study of MAPbI_3_ and MAPbCl_3_, along with their lead halide precursors PbI_2_ and PbCl_2_. In order to understand the interface effects between MAPbI_3_ and MAPbCl_3_, a third sample composed by both the perovskites has been prepared. We used X-ray diffraction to confirm the high quality and phase purity of films, and photoemission spectroscopy to study their electronic properties. From Lead core-level photoemission we gain insight in the chemistry of the various compounds, while UV-excited valence bands provide information on band alignment and charge transfer mechanisms. Finally, photoemission spectra have been compared with first-principles GW calculations.

## Results and Discussion

Lead halide samples have been evaporated *in situ* in high vacuum (P < 10^−8^ mbar) on a polycrystalline gold sample for photoemission experiments, while diffraction measurements have been performed on powder crystallized on a nylon loop. MAPbI_3_ sample has been prepared by spin-coating a pure iodine perovskite solution (CH_3_NH_3_I and PbI_2_ 1:1 in DMF) on silicon < 100 > wafers (20 × 20 mm^2^) (2000 rpm for 60 seconds). Substrates were placed on a hot plate for 5 minutes at 85 °C to achieve complete evaporation of solvent and crystallization of the hybrid organic-inorganic material. MAPbCl_3_ sample for photoemission experiment has been prepared with a variation of the two-step method: PbCl_2_ was evaporated on a polycrystalline gold substrate, and a saturated methylammonium chloride (MACl) solution in ethanol was drop-casted on the obtained film. Diffraction measurement has been performed crystallizing a solution of MACl and PbCl_2_ 1:1 in DMSO on a nylon loop without subsequent annealing. The mixed perovskite phase has been obtained evaporating PbI_2_ on polycrystalline gold (for the photoemission measurements) or silicon on a kapton tape (for the diffraction measurements) and drop-casting a solution of MACl and PbI_2_ 1:1 5% in weight in DMF. All the drop-casting processes have been performed with the sample placed on a hot plate kept at 80 °C up to the complete evaporation of the solvent.

**Figure 1:**
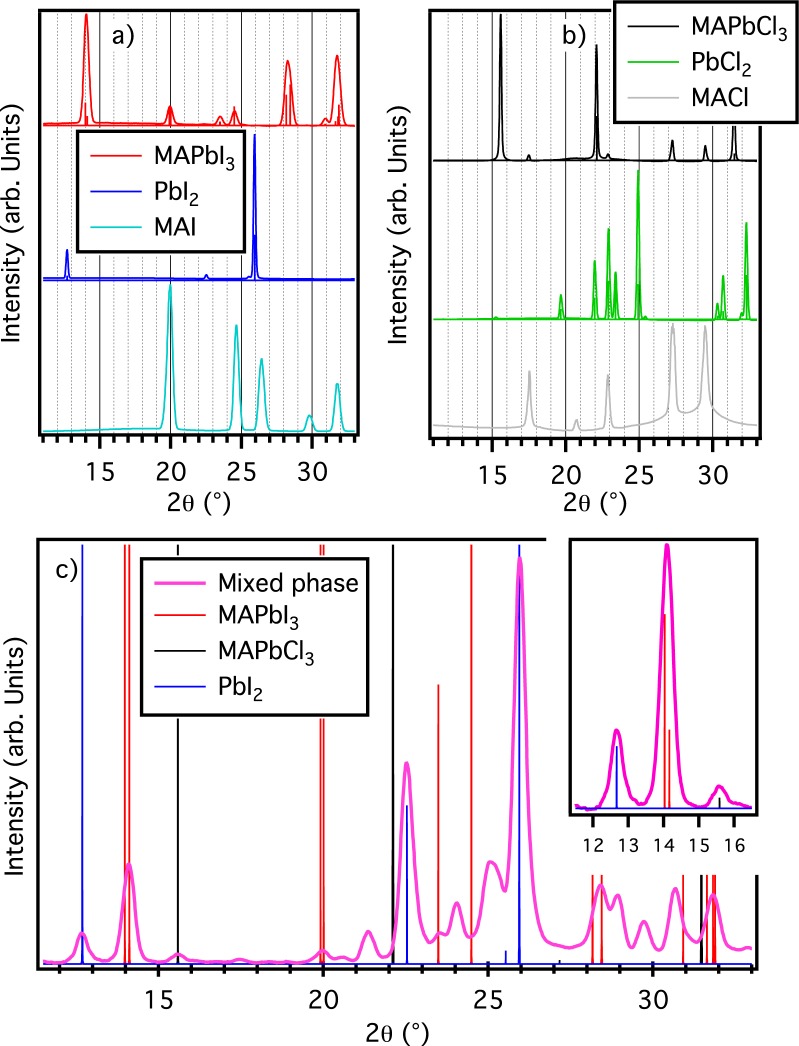
Diffraction results. Excerpts from diffraction patterns for MAPbI_3_ (red), PbI_2_ (blue), and MAI (cyan) in panel (a). Diffraction patterns for MAPbCl_3_ (black), PbCl_2_ (green), and MACl (grey) in panel (b). Diffraction patterns for mixed perovskite (purple), MACl (gray) in panel c). Vertical bars in all panels indicate the position of MAPbI_3_ (red), MAPbCl_3_ (black), and PbI_2_ (blue) peaks according to calculations. All the patterns are reported using a Cu Kα wavelength (1.5418 Å), for direct comparison among data collected at different wavelengths and literature data (complete raw data are reported in Fig. S1).

XRD characterization has been performed to identify the phases present in all the synthesized samples. The lattice parameters of the perovskite phases have been refined and agree with literature data (Table S1). Figure 1 shows the powder diffraction pattern of all the investigated samples. Panels a) and b) show the diffraction pattern of tetragonal MAPbI_3_ and cubic MAPbCl_3_ perovskites, along with the diffraction patterns of the stock reagents used in the synthesis, respectively PbI_2_, MAI, PbCl_2_, and MACl. Upon formation of MAPbI_3_ perovskite by spin coating process no traces of residual of PbI_2_ or MAI can be detected, while panel b) shows that residual traces of MACl remains (peaks at 17.5, 22.9, 27.2, 29.5 degrees) in the MAPbCl_3_ formation process when deposition is not followed by an annealing. These data definitely support the importance of a mild annealing after the perovskite formation. The two-step process therefore does not ensure the formation of a pure perovskite sample, leaving a certain amount of unreacted precursor, mainly methylammonium compounds. An annealing performed in the temperature range 80–100 °C is enough to remove the metyhlammonium residual without ruining the perovskite film^21,22^.

Panel c) of Fig. 1 shows the diffraction pattern of the mixed perovskite sample once the background coming from the kapton tape has been removed. Majority of the diffraction signals can be associated to two perovskites, along with a certain amount of unreacted reagents (PbCl_2_, PbI_2_, MAI) and a mixed lead halide phase (PbClI). Curve fitting of the whole profile, reported in Supplementary Fig. S2, has been obtained using Le Bail refinement (Rwp of 1.3%). Inset shows the region 12–16°: here we can easily distinguish the contribution coming from PbI_2_ (12.7°), MAPbI_3_ (14.1°), and MAPbCl_3_ (15.6°). Supplementary Materials report crystallographic parameters of all the phases found (Table S2).

**Figure 2:**
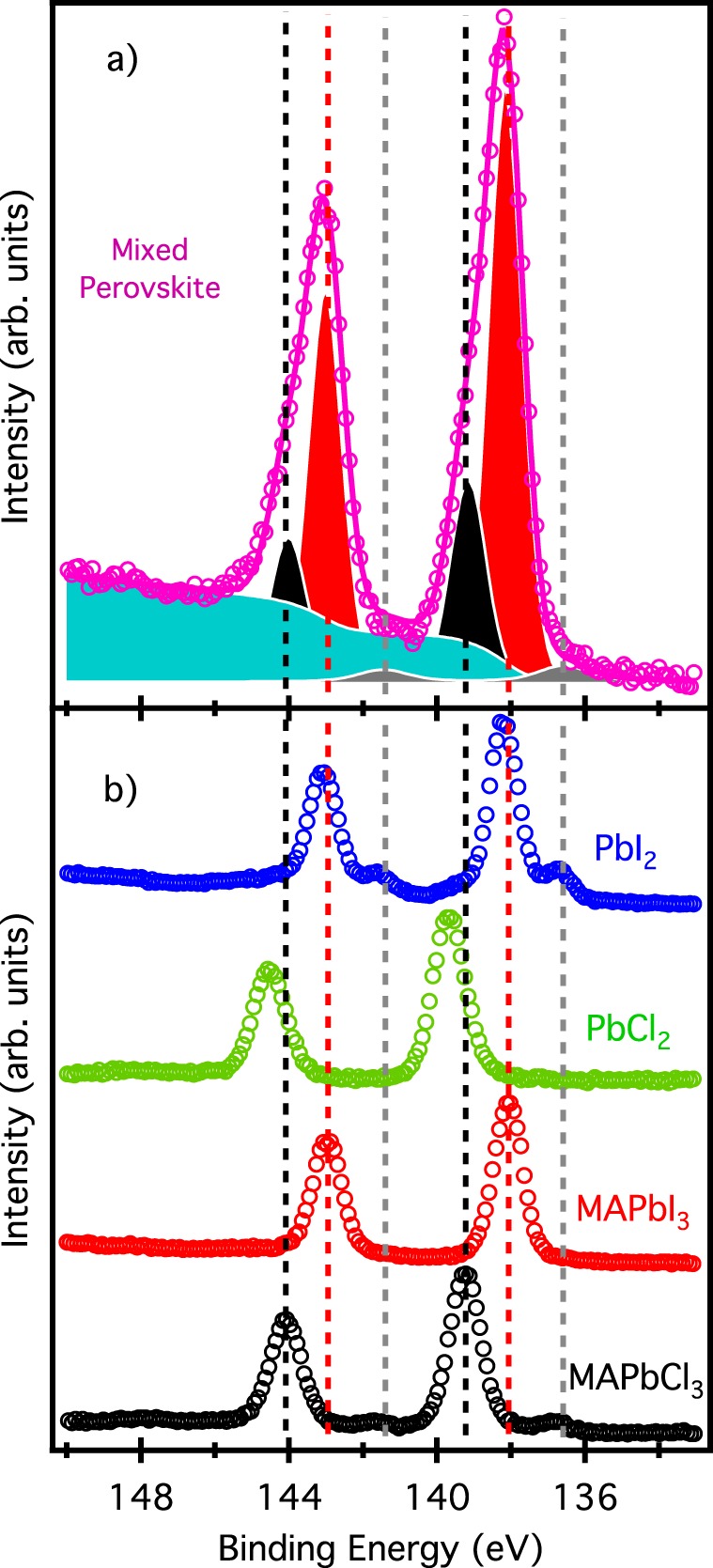
Pb 4 f core level. Spectra of the mixed perovskite grown on PbI_2_/Au (purple circles, panel a), and the benchmarks MAPbCl_3_/PbCl_3_/Au (black circles, panel b), MAPbI_3_/Si (red circles, b), PbCl_2_/Au (green circles, b), PbI_2_/Au (blue circles, b) acquired with a photon energy of 280 eV. The fit result is also reported for the mixed perovskite sample: gray peaks correspond to elemental Pb (Pb^0^), red peaks to MAPbI_3_ component, and black peaks to MAPbCl_3_ one. The cyan component is the background due to the inelastically scattered electron during the photoemission process. Vertical bars represent the energy of the benchmark peaks.

After a complete solid phase characterization of our samples have been obtained, photoemission spectroscopy has been exploited to determine their electronic structure. Figure 2b shows the Pb 4 f photoemission peaks of our reference compounds MAPbCl_3_, MAPbI_3_, PbCl_2_, and PbI_2_. All these spectra, besides the PbCl_2_ one, show a low energy component at 136.7 eV: this corresponds to interstitial elemental Pb^10,24,36^. It is worth noticing that the energy of the two lead salt peaks is remarkably different (1.5 eV) even if they are chemically very similar bearing formally the same lead oxidation state. The electron affinity of chlorine is in fact higher than the iodine one, controlling the charge transfer from lead atoms. In particular, an increase of the electron affinity of the halide corresponds to an increase of the binding energy of the lead peaks. Upon perovskite formation we can see that both MAPbCl_3_ and MaPbI_3_ show a lower Pb 4 f binding energy compared to their respective lead halide salts equivalents (less than 0.1 eV for PbI_2_ - MAPbI_3_, and 0.6 eV for PbCl_2_ - MAPbCl_3_), suggesting an effective total energy lowering induced by the perovskite formation, even more pronounced in the MAPbCl_3_ case.

The mixed perovskite shows much more broad and asymmetric lead peaks, indicating the presence of different and well-separated components. We fitted these peaks with three voigt components and a Shirley background (shown in Fig. 2a): the first component has been held at 136.7 eV to take in account for the elemental lead component, the other two left free to move. The fit result is reported in Table 1: the energy of the two free components shows an excellent similarity with the energy of lead peaks in pure MAPbCl_3_ and MAPbI_3_ perovskites. These results imply that little, or none, band bending occurs at the MAPbCl_3_/MAPbI_3_ interface. From this analysis the mixed perovskite sample results to be formed by 29% of MAPbCl_3_ and 71% of MaPbI_3_.

**Table 1:** Pb 4f_7/2_ binding energy for MAPbCl_3_ and MaPbI_3_.

	Lead binding energy (MaPbI_3_ component)	Lead binding energy (MAPbCl_3_ component)
MaPbI_3_ Perovskite	138.08 ± 0.20 eV	—
MAPbCl_3_ Perovskite	—	139.05 ± 0.07 eV
Mixed Perovskite	138.25 ± 0.23 eV	139.05 ± 0.23 eV

The last row is the fitting result for the mixed perovskite.

Since the binding energy of lead in PbI_2_ is very similar to the one in MAPbI_3_, it can be argued that the peak assigned to the perovskite could be a mixture of the two. This conclusion seems confirmed by XRD data, which show the presence of also lead halide. However, we have to consider the different probing depth of the two techniques: XRD is extremely bulk sensitive since it is a photon-in photon-out technique, while exactly the opposite holds for soft x-rays and UV photoemission. The kinetic energy of the analyzed electron in this experiment (both core levels and valence band) is in the range 50–150 eV, with an inelastic mean free path of less than 1 nm. For this reason, it is more consistent to compare core level with valence band data that, as we will discuss in the following, do not give us any evidence of PbI_2_ on the surface of the mixed sample. We can conclude that most of the PbI_2_ seen in the diffraction pattern comes from the substrate/perovskite interface, were a thick film of lead iodide has been evaporated.

**Figure 3:**
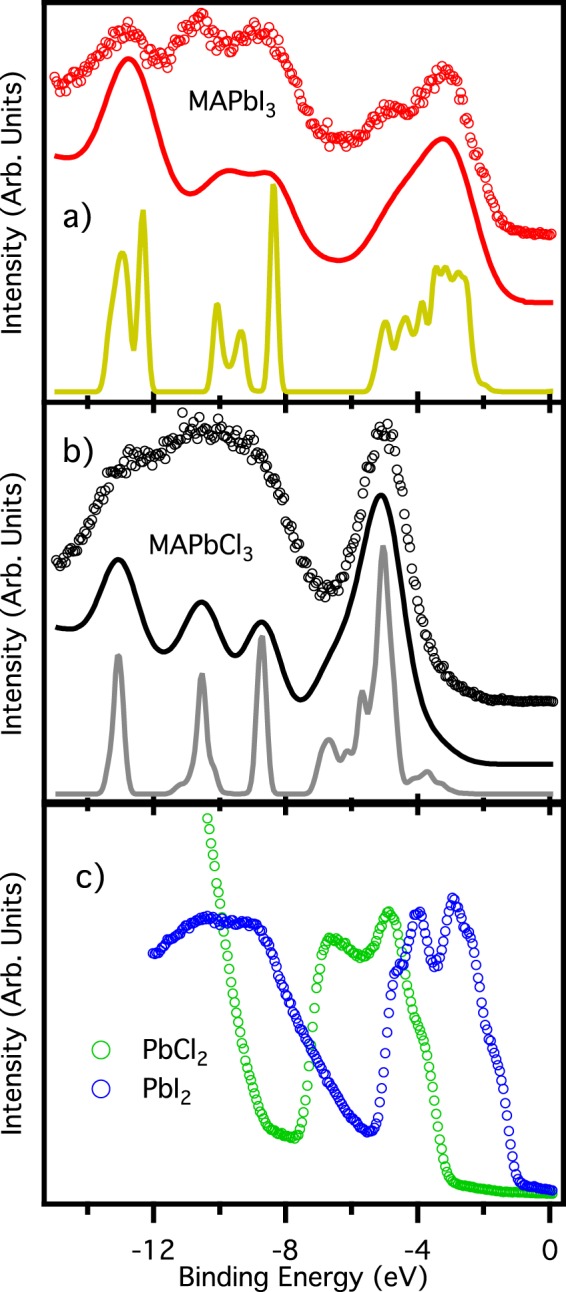
Valence band. Spectra of MAPbI_3_/Si (panel a), and MAPbCl_3_/PbI_3_/Au (**b**), and PbI_2_/Au and PbCl_2_/Au (**c**). For each panel the bottom continuous line is the GW calculated spectrum, while the top continuous line is the same spectrum convoluted with a gaussian function of 200 meV FWHM to take into account the finite experimental resolution, and with the addition of a Shirley background. Circles represent the experimental points. Valence bands in panel c are acquired with photon energy of 21.22 eV (He I line), while all the others valence bands are acquired with photon energy of 55 eV.

Finally, Fig. 3 shows the valence band of the two perovskites (panels a and b) along with lead iodide and chloride salts (panel c). These spectra are acquired with low energy photons (55 eV), differently from most of the other perovskite valence bands published up to now^10,24,33,36,37^. The low photon energy we used ensures a comparable photoemission cross section for lead/iodine and carbon/nitrogen derived states, permitting us to highlight the structure in the 6–12 eV binding energy range. Considering that the calculated DOS does not take into account the different cross section of the different element-derived states, we can conclude that accordance between experimental data and calculations is fairly good. This remarks once again the excellent quality of our samples, comparable with *in-situ* cleaved ones^38^, that permits us to determine with a high degree of reliability the energy level alignment of the perovskites films.

Samples with such high-quality surface do not need the more bulk sensitive high-energy photons for a clear determination of the valence band maximum, with the advantage of gaining energy resolution. Moreover, it is worth noting that none of our valence bands show an appreciable Fermi level, regardless the amount of metallic lead appearing in XPS spectra.

**Figure 4:**
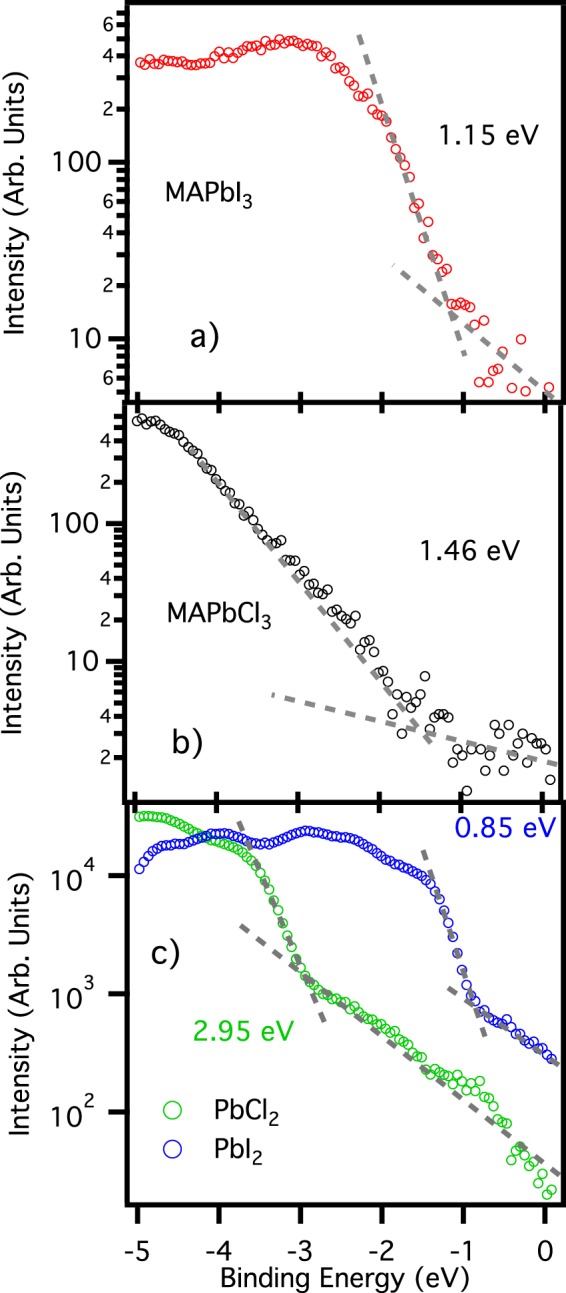
Valence band maximum determination. Spectra of Fig. 3 enlarged in the region near the valence band maximum and plotted on a semi-logarithmic scale. Valence band maximum is calculated as the energy of the crossing point between the extrapolation of the background and the extrapolation of the valence band onset.

Figure 4 shows an enlarged view of the photoemission spectra near the valence band maximum plotted in a logarithmic scale graph, used to extract the value of the valence band maximum (in Supporting Information the same procedure using linear scale graphs). The measured value of 1.15 eV for the valence band maximum of MAPbI_3_ is in fair accordance with the previously reported values, confirming that this compound is a n-doped semiconductor. The MAPbCl_3_: looks instead an intrinsic semiconductor, with its band gap of 2.9 eV, and its valence band maximum placed at 1.46 eV.

**Figure 5:**
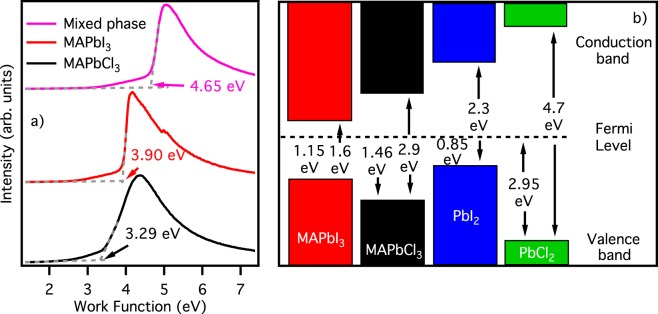
Energy level alignment. Panel (a) secondary electron cut-off for MAPbI_3_, MAPbCl_3_, and the mixed phase. The energy scale has been already converted to work function value (referred to Fermi level) taking into account bias and photon energy. Panel (b) energy level scheme for MAPbI_3_, MAPbCl_3_, and the two lead halide salts. Spaces are not in scale.

Panel b of Fig. 5 summarizes the energy level position derived from our data, joined with band gap values found in literature^2,22,30,37,39,40^. In this scheme bands are aligned to the Fermi level, as it would be in the case of an interface, but far enough from it not to take into account any band bending effect. From this scheme it is evident that the only interface capable of promoting the charge transfer from the MAPbI_3_ is the MAPbI_3_/PbI_2_ one. In fact, at this interface holes can be transferred from MAPbI_3_ to PbI_2_, but not electrons. While valence band maximum of PbI_2_ is approximately 0.3 eV closer to the Fermi level with respect to the MAPbI_3_ one, and hence capable of accepting holes, its conduction band is 1 eV further away from Fermi, forbidding any electron transfer from MAPbI_3_ to PbI_2_. This scheme is consistent with some others presented in the literature^41,42^, but differs from the one presented by Chen *et al*.^43^. In the latter, the authors claim that the presence of PbI_2_ between the perovskite absorber and the electron transfer material TiO_2_ helps to avoid recombination between the electron injected in the scaffold and the remaining hole in the perovskite, but on the basis of our data we can exclude that any electron transfer from the perovskite to TiO_2_ (or any other electron transporting material) occurs *via* a PbI_2_ layer. From our scheme it results also clear that no charge transfer, neither of electrons nor of holes, is possible from MAPbI_3_ to MAPbCl_3_.

**Figure 6:**
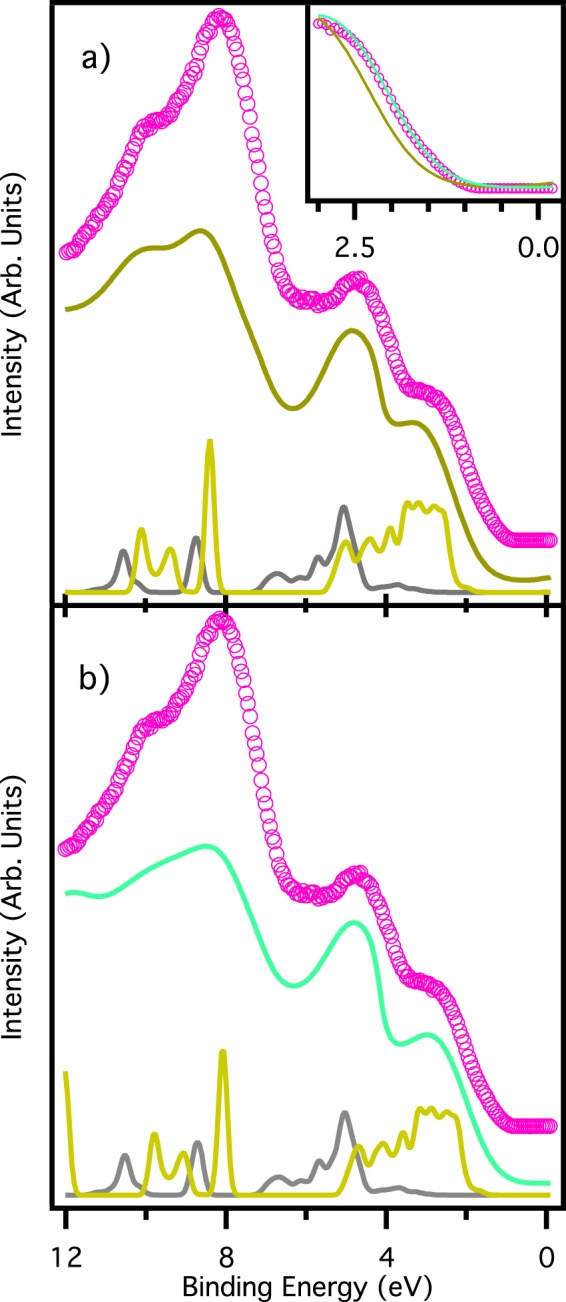
Mixed perovskite. Valence band of mixed perovskite grown on PbI_2_/Au (purple circles in both panels), along with two different calculations as weighted sum of the valence bands of MAPbI_3_ and MAPbCl_3_. In panel (b) the contribution of MAPbI_3_ is shifted by 0.3 eV towards lower binding energies. In the inset the enlarged view of the valence band maximum with experimental spectrum and both calculated spectra.

The valence band of the mixed perovskite shows a peculiar behavior. Figure 6 shows the experimental UPS spectrum (purple circles) acquired with a photon energy of 21.22 eV (He I emission line), along with two differently displaced calculated spectra. It is worth noticing that the photon energy used to acquire this valence band spectrum is different from the previous one (21.22 vs 55 eV), however, the change in probing depth and difference in photoionization cross section do not affect the value of the quantity we are mostly interested in, i.e. the valence band maximum. In both panels, the experimental spectrum is compared with a total DOS formed by simply adding the calculated DOSs of MAPbI_3_ and MAPbCl_3_. Once again accordance between the experimental spectrum and the calculated DOS is reasonable, meaning that basically MAPbI_3_ and MAPbCl_3_ form two distinct phases in the sample, with no interplay between them.

In panel a, each single calculated DOS has been placed at the same binding energies of the calculated DOSs in panels a and b of Fig. 3. This choice ensures a perfect energy matching between total DOS and experimental spectrum for the peak at 5.5 eV of binding energy, whose major contribution comes from the MAPbCl_3_ DOS. However, the same does not hold for the structures between 1 and 3 eV and at 8 eV of binding energy, mainly due to MAPbI_3_ DOS. Moreover, the valence band onset is not well matched in this condition, as highlighted in the inset of Fig. 6: calculated DOS (bronze continuous line) is shifted 0.3 eV toward higher binding energies with respect to the experimental spectrum (violet circles). To overcome these problems a new total DOS has been shaped: in panel b of Fig. 6 it is shown as a green continuous line. This new total DOS is again the sum of the single MAPbI_3_ and MAPbCl_3_ DOSs, but in this case the MAPbI_3_ DOS has been shifted by 0.3 eV towards lower binding energies with respect to its original position. This new total DOS still matches the peak at 5.5 eV, but now is capable of matching well also the region at 8 eV and the valence band onset, as shown in the inset of Fig. 6. The shift of the MAPbI_3_ component of the valence band is also reflected by the valence band maximum: in this mixed sample it is 0.86 eV as extrapolated once again from a logarithmic plot, i.e. 0.3 eV closer to the Fermi level with respect to the pure MAPbI_3_ perovskite.

In both cases the total DOS has been composed with 30% of MAPbCl_3_ and 70% of MaPbI_3_ calculated DOSs, consistently with the amount deduced by the XPS data. The accordance between experimental data and calculations is good even without adding PbI_2_ derived features, confirming that PbI_2_ is underneath the perovskite film as discussed for XPS data, too deep to be detected by photoemission. To corroborate this hypothesis, we can notice that the valence band offset for this mixed phase (inset of Fig. 6) is around 100 meV shifted toward higher binding energy with respect to the PbI_2_ onset (Fig. 4c), excluding definitely the possibility that photoemission spectra for this sample show any PbI_2_ related features.

The shift of the MAPbI_3_ valence band looks really similar to what observed by Wang *et al*., where a perovskite self-doping is observed just due to a growth procedure that uses an excess of MAI^29^. This is also supported by the electron secondary cuts reported in panel (a) of Fig. 5. Here, we find the work function values for MAPbI_3_ and MAPbCl_3_ perovskites, which are in good accordance with the already known value, and for the mixed perovskite we prepared. We can see that, the while valence band is the superposition of the two contributions of MAPbI_3_ and MAPbCl_3_, the work function neither is intermediate between the two, nor the lowest one. Exactly as in the case of a MAPbI_3_ perovskite growth with an excess of MAI, the work function of our mixed perovskite is higher than the stoichiometric one. However, in our growth process there is no excess of MAI, but rather a lack. We drop casted a solution 1/1 PbI_2_:MAI, i.e. a stoichiometric solution, on a PbI_2_ film, and XRD patterns and photoemission spectroscopy spectra suggest that our sample is essentially a stoichiometric (even if mixed) perovskite deposited on PbI_2_ film. On the other hand, panel b of Fig. 5 shows clearly that the Fermi level of PbI_2_ is not in the center of its band gap, but rather closer to the valence band maximum, as the case of a p-type semiconductor. This picture is closer to the one described by Miller *et al*., where MAPbI_3_ deposited on different p and n-type semiconductor shows a different doping level^33^. Finally, it is worth noting that the MAPbCl_3_ valence band is perfectly aligned between the pure and the mixed phase, meaning that whatever is the origin of doping in MAPbI_3_, this does not affect the MAPbCl_3_ perovskite.

## Conclusion

In this paper we have investigated the electronic structure of MAPbI_3_, MAPbCl_3_, and their lead halide precursors PbI_2_ and PbCl_2_. High resolution photoemission spectra have been compared with accurate GW calculations. The good accordance between experimental spectra and calculations, along with XRD, confirm the high reliability of our data, allowing us to be highly confident on the correctness of the extracted information.

Valence band alignment between perovskites and lead halide precursors clearly indicates that no charge transfer between them is possible, except for a hole transfer process from MAPbI_3_ to PbI_2_.

A third perovskite sample, composed by both MAPbI_3_ and MAPbCl_3_ has also been investigated by means of X-ray diffraction and photoemission. Here a different doping level for the MAPbI_3_ component has been found, possibly related to the p-type nature of the PbI_2_ film forming the substrate of the perovskite.

Best efficiency performances have been obtained by a smart design of the heterojunctions for charge transfer^44^, demonstrating the importance of a precise knowledge of the electronic structure of all the components. The high accuracy electronic structure determination we performed will therefore provide robust basis for further boosting of devices performance. Always in this framework, however, future studies on the doping origin of perovskite are required, as the ability of tuning this doping level can add a new parameter in devices design.

## Methods

### Photoemission experiments

Photoemission experiment has been performed in a modified VG Escalab MKII (secondary cut-off electrons and valence bands at 21.22 eV) and on BEAR beamline at Elettra^45^ (core levels and valence bands at 55 eV). The overall resolution is 200 meV for valence band spectra and 800 meV for core level spectra. All binding energies are referred to the Fermi level of the spectrometer. Binding energy calibration has been performed using the elemental Pb4f_7/2_ (Pb^0^) component present in all the spectra for core levels, while valence bands have been aligned using a gold foil placed in the vicinity of the sample.

### Diffraction experiments

Diffraction experiments have been performed at room temperature at the XRD1 beamline at Elettra^46^. The powder diffraction patterns of MAPbI_3_ and mixed perovskites sample (both grown on thick substrates) have been collected in transmission mode using a monochromatic wavelength of 0.7 Å with an exposure time of 30 seconds, while the perovskite sample MAPbCl_3_ (mounted on nylon loop) has been collected using a monochromatic wavelength of 1 Å for 60 seconds (to improve count statistics). Bidimensional diffraction patterns were recorded on a PILATUS 2 M hybrid pixel area detector (pixel dimension 172 μm - Dectris Ltd., Baden-Daettwil, Switzerland) at a distance of 200 mm for MAPbCl_3_ and 85 mm for MAPbI_3_ and mixed perovskite phase. One-dimensional diffraction patterns (Fig. S2a–c) were obtained in the 2θ range 5−25° for both MAPbI_3_ and mixed MAPbI_3_/MAPbCl_3_ samples and in the 2θ range 7−47° for MAPbCl_3_ sample by integrating the two-dimensional images with the program FIT2D. Standard experimental calibration has been followed using a 0.3 mm borosilicate capillary filled with NIST LaB6 660a standard powder (SRM660), to define the beam centre, adjust detector distance and tilting. Monochromator energy has been calibrated using a fluorescence scan at selenium K absorption edge. All the experimental patterns have been analyzed using a whole powder pattern fitting (Le Bail method) using the GSAS Package with the EXPGUI interface. All the phases used to model the synthetized compounds are listed in the Table S2 of the SI.

### Calculations

DOSs have been obtained using the GW approximation in which the electron self-energy is expressed as the product of the electron one-body Green’s function (G), evaluated at the Density Functional Theory (DFT) level, with the screened Coulomb interaction (W) which in turn is evaluated within the random phase approximation^47,48^. Spin-orbit coupling (SOC) is pivotal for the correct assessment of electronic properties^49^. Calculations were performed using the Quantum-Espresso^50,51^ suite of DFT packages. The generalized gradient approximation (GGA) for the exchange and correlation energy described in ref.^52^ is used in the DFT calculations. We investigated the room-temperature tetrahedral phases of MAPbI_3_ and MAPbCl_3_ involving a primitive cell comprising 48 atoms, which is described through periodic boundary conditions and discrete sampling of the Brillouin zone. Both lattice parameters and atomic positions have been optimized at the DFT level. A thorough description of the technical details required by our SOC-GW calculations is reported in refs^49^.

Data are available upon request to the corresponding authors.

## Supplementary information

Supplementary Info
