# Supplementary material for: Electronic structure of MAPbI3 and MAPbCl3: importance of band alignment
Source: Sci Rep. 2019 Oct 22;9:15159. doi: 10.1038/s41598-019-50108-0 (PMC6805902; doi:10.1038/s41598-019-50108-0)
Supplement: Supplementary file 1 — Supplementary Info [file 41598_2019_50108_MOESM1_ESM.doc]

Table S1. Space Group and unit cell parameters of the MaPbI3 and MAPbCl3 samples compared with the literature data.

|  | **LeBail refined values** | |  | **Literature values** | |
| --- | --- | --- | --- | --- | --- |
| **Sample** | **Space Group** | **Cell parameters** | ***Rwp*** | **Space**  **Group** | **Cell parameters** |
| **MAPbI_3_**  (Figure 1a) | *I* 4*cm* | *a*=*b*=8.849(2)  *c*=12.633(1)  *α*=*β*=*γ*=90° | 0.64% | *I* 4*cm*^*^ | *a*=*b*=8.849(2)  *c*=12.642(2)  *α*=*β*=*γ*=90° |
| **MAPbCl_3_**  (Figure 1b) | *P m*-3*m* | *a*=*b*=*c*=5.680(2)  *α*=*β*=*γ*=90° | 0.74% | *P m*-3*m*^**^ | *a*=*b*=*c*=5.675(2)  *α*=*β*=*γ*=90° |
| ^*^ Constantinos C. Stoumpos, Christos D. Malliakas, and Mercouri G. Kanatzidis Semiconducting Tin and Lead Iodide Perovskites with Organic Cations: Phase Transitions, High Mobilities, and Near-Infrared Photoluminescent Properties Inorg. Chem. 2013, 52, 9019−9038  ^**^ Kawamura, Y.; Mashiyama, H. Modulated Structure in Phase II of CH_3_NH_3_PbCl_3_. J. Korean Phys. Soc.1999, 35, 1437. | | | | | |

Table S2. Space Group and unit cell parameters of all the phases found in the samples reported in this work.

| **Phases** | **Space Group** | **Ref** | **Cell parameters (Å, °)** |
| --- | --- | --- | --- |
| **MAPbI_3_** | *I* 4*cm* | Constantinos C. Stoumpos, Christos D. Malliakas, and Mercouri G. Kanatzidis, Semiconducting Tin and Lead Iodide Perovskites with Organic Cations: Phase Transitions, High Mobilities, and Near-Infrared Photoluminescent, Properties Inorg. Chem. 2013, 52, 9019−9038 | *a*=*b*=8.849(2)  *c*=12.633(1)  *α*=*β*=*γ*=90 |
| **MAPbCl_3_** | *P m*-3*m* | Kawamura, Y.; Mashiyama, H., Modulated Structure in Phase II of CH3NH3PbCl3, J. Korean Phys. Soc.1999, 35, 1437. | *a*=*b*=*c*=5.6804(2)  *α*=*β*=*γ*=90 |
| **MAI** | *P bma* | [O. Yamamuro](http://scripts.iucr.org/cgi-bin/citedin?search_on=name&author_name=Yamamuro, O.), [T. Matsuo](http://scripts.iucr.org/cgi-bin/citedin?search_on=name&author_name=Matsuo, T.), [H. Suga](http://scripts.iucr.org/cgi-bin/citedin?search_on=name&author_name=Suga, H.), [W. I. F. David](http://scripts.iucr.org/cgi-bin/citedin?search_on=name&author_name=David, W.I.F.), [R. M. Ibberson](http://scripts.iucr.org/cgi-bin/citedin?search_on=name&author_name=Ibberson, R.M.) and [A. J. Leadbetter](http://scripts.iucr.org/cgi-bin/citedin?search_on=name&author_name=Leadbetter, A.J.), Neutron diffraction and calorimetric studies of methylammonium iodide, Acta Cryst. (1992). B48, 329-336 | *a*=7.174  *b*=7.097  *c*=8.832  *α*=*β*=*γ*=90 |
| **MACl** | *P* 4*/nmm* | Edward W. Hughes, William N. Lipscomb, The Crystal Structure of Methylammonium Chloride,J. Am. Chem. Soc., 1946, 68 (10), pp 1970–1975 | *a*=*b*=6.04  *c*=5.05  *α*=*β*=*γ*=90 |
| **PbCl_2_** | *P bnm* | R.W.G. Wyckoff, Crystal Structures, John Wiley, New York (1963) | *a*=9.030  *b*=7.608  *c*=4.525  *α*=*β*=*γ*=90 |
| **PbI_2_** | *P* -3*m*1 | R.W.G. Wyckoff, Crystal Structures, John Wiley, New York (1963) | *a*=*b*=4.555  *c*=6.977  *α*=β=90  *γ*=120 |
| **PbClI** | *P nam* | L.H. Brixner, H.-Y. Chen, C.M. Foris, X-ray study of the PbClI and PbBrI systems, Journal of Solid State Chemistry, Volume 40, Issue 3, 1981, Pages 336-343 | *a*=8.187  *b*=9.654  *c*=4.593  *α*=*β*=*γ*=90° |
| * LeBail refined values are reported, using data reported in this work | | | |

Figure S1. Experimental powder patterns of a) MAPbCl_3_, b) MAPbI_3_ and c) mixed MAPbCl_3_/ MAPbI_3_

Figure S2. Experimental mixed perovskite sample XRPD pattern (red cross), calculated powder pattern (green line) and corresponding residuals (purple line) from Le Bail refinement. Reported ticks correspond to identified phases: MAPbCl_3_ (magenta), MAPbI_3_ (yellow), PbCl_2_ (green), PbI_2_ (blue) MAI (brown) and PbClI (black)


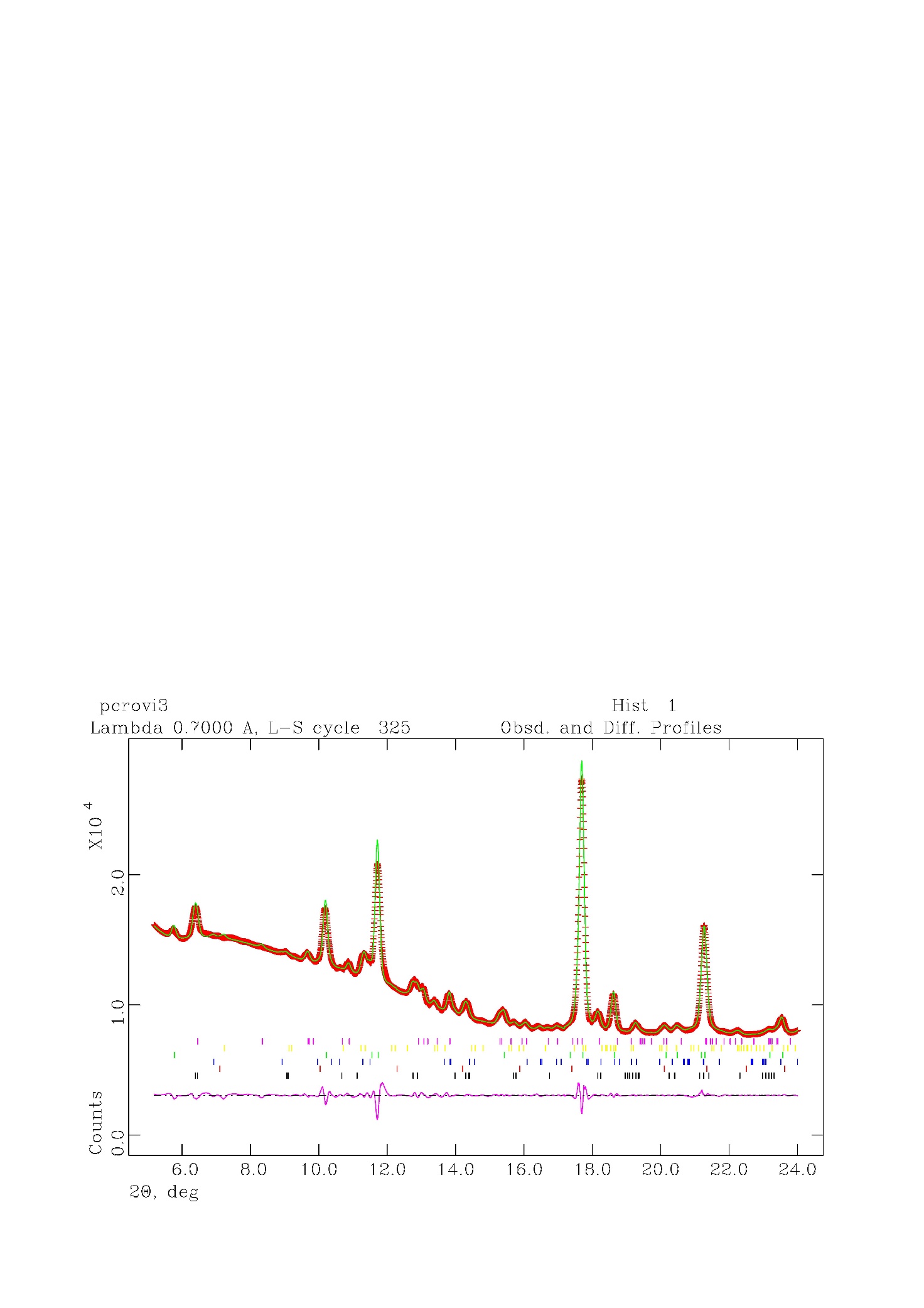


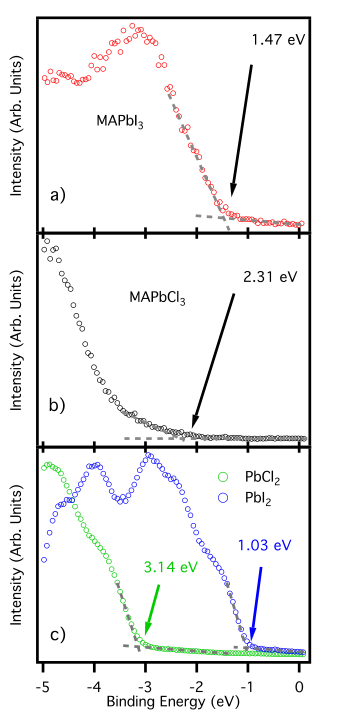


Figure S3. Spectra of figure 3 of the main text enlarged in the region near the valence band maximum. The valence band maxima reported here are calculated using linear extrapolation.


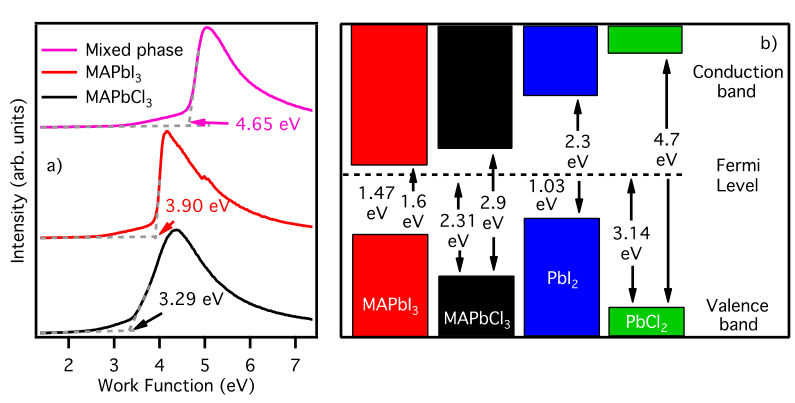


Figure S4. Panel a: secondary electron cut-off for MAPbI_3_, MAPbCl_3_, and the mixed phase. The energy scale has been already converted to work function value (referred to Fermi level) taking into account bias and photon energy. Panel b: energy level scheme for MAPbI_3_, MAPbCl_3_, and the two lead halide salts calculated using linear extrapolation. Spaces are not in scale.
